# Diagnostic Approaches for Invasive Aspergillosis—Specific Considerations in the Pediatric Population

**DOI:** 10.3389/fmicb.2018.00518

**Published:** 2018-03-26

**Authors:** Thomas Lehrnbecher, Angela Hassler, Andreas H. Groll, Konrad Bochennek

**Affiliations:** ^1^Division of Pediatric Hematology and Oncology, Hospital for Children and Adolescents, Johann Wolfgang Goethe-University, Frankfurt, Germany; ^2^Infectious Disease Research Program, Center for Bone Marrow Transplantation, Department of Pediatric Hematology/Oncology, University Children's Hospital, Münster, Germany

**Keywords:** *Aspergillus*, child, cancer, diagnostics, galactomannan, ß-D-glucan, polymerase chain reaction, imaging

## Abstract

Invasive aspergillosis (IA) is a major cause of morbidity and mortality in children with hematological malignancies and those undergoing hematopoietic stem cell transplantation. Similar to immunocompromised adults, clinical signs, and symptoms of IA are unspecific in the pediatric patient population. As early diagnosis and prompt treatment of IA is associated with better outcome, imaging and non-invasive antigen-based such as galactomannan or ß-D-glucan and molecular biomarkers in peripheral blood may facilitate institution and choice of antifungal compounds and guide duration of therapy. In patients in whom imaging studies suggest IA or another mold infection, invasive diagnostics such as bronchoalveolar lavage and/or bioptic procedures should be considered. Here we review the current data of diagnostic approaches for IA in the pediatric setting and highlight the major differences of performance and clinical utility of the tests between children and adults.

## Introduction

Invasive fungal disease (IFD), in particular invasive aspergillosis (IA), is a major cause of morbidity and mortality in immunocompromised children and adults (Pagano et al., 2007; Sung et al., 2007; Upton et al., 2007). The definite diagnosis of IA (“proven aspergillosis”) is extremely difficult and requires the visualization of the organism in tissue or its microbiological detection in an otherwise sterile compartment. In daily clinical practice, the diagnosis of IA is mostly made by appropriate imaging studies and diagnostic biomarkers in blood and other readily available specimens (“probable or possible aspergillosis”), and these methods are important guiding decisions about the institution and the choice of antifungal agents as well as regarding the duration of therapy. The rationale for this clinical practice is the fact that early institution of appropriate antifungal agents is the factor that is most strongly associated with favorable outcome (Caillot et al., 1997).

Different strategies have been developed in order to decrease the incidence of IA and to improve outcome. Many experts in the field administer prophylactic mold-active antifungals to patients at high-risk for IA. In the pediatric setting, this population includes patients with acute myeloid leukemia (AML), relapsed acute leukemia [both AML and acute lymphoblastic leukemia (ALL)] as well as children undergoing allogeneic hematopoietic stem cell transplantation (HSCT), all of which have a risk >10% for IA (Sung et al., 2007; Hol et al., 2014; Fisher et al., 2017). The situation is more complex in children undergoing therapy for ALL, the largest patient population in pediatric hematology. Depending on the protocol used and some additional factors such as age, the presence of prolonged bone marrow failure, and concomitant use of corticosteroids, some of these patients are at high risk for IA, whereas others are at low risk; unfortunately, however, these subgroups are poorly defined to date (Groll et al., 2014). In contrast, children undergoing autologous HSCT and children diagnosed with non-Hodgkin and Hodgkin lymphoma as well as children with solid tumor have a low risk for IA, although IA is sometimes seen in these patients (Groll et al., 2014). Some institutions may not use antifungal prophylaxis but screen afebrile and asymptomatic neutropenic patients at high-risk for IA using non-culture based methods, and institute mold-active compounds only if the patient develops fever and screening tests turn positive (“pre-emptive therapy”). However, pediatric data of pre-emptive therapy are scarce, and therefore, in children, the approach to use empirical antifungal therapy is more common. With this concept, antifungals are started in all high-risk neutropenic patients who develop fever which does not respond to broad spectrum antibiotics.

The result of a diagnostic tool is not only important in the decision to start antifungal therapy, but may also help to modify and guide the use of antifungals. Although, as compared to studies with adult patients, considerably less data are available in the pediatric population, it has been recognized that the performance and the usefulness of diagnostic tools to detect IA may differ between children and adults. This has led to several pediatric specific guidelines for the diagnosis and treatment of IFD (Groll et al., 2014; Science et al., 2014; Lehrnbecher et al., 2017b), and at present, the European Organisation for Research and Treatment of Cancer (EORTC) and the Mycosis Study Group (/MSG) (EORTC/MSG) consensus definitions for the diagnosis of IFD undergo a second revision in which pediatric specific diagnostic considerations will be included. The present review will focus on the value of diagnostic tests to detect IA in the pediatric setting and highlight major differences between children and adults.

## Clinical signs and symptoms of invasive aspergillosis

Similar to adults, clinical signs, and symptoms of IA in children are often non-specific and cannot be readily distinguished from other infections caused by bacteria, viruses, and other fungi or from certain non-infectious complications caused by the underlying disease or sequelae of its treatment. Importantly, fever during neutropenia which is unresponsive to broad-spectrum antibiotics may be the only sign of IA. Other clinical symptoms depend of the site of infection: as IA most often involves the lung, patients may complain about pleural or back pain or dyspnea, whereas headache, altered mental state, seizure, or focal neurologic signs may indicate central nervous system (CNS) involvement (Hassler et al., 2015; Lehrnbecher et al., 2017a).

## Microscopy and fungal culture

Overall, standard diagnostic techniques such as microscopy and culture to detect fungal organisms do not differ between children and adults. A microbiologist experienced in the diagnosis of fungal infections should microscopically examine all samples from invasive diagnostic procedures such as bronchoalveolar lavage (BAL) or biopsies (Ruhnke et al., 2012). Special “fungal stains” such as periodic acid—Schiff, Grocott's methenamine silver, or optical brighteners (e.g., calcofluor white) are recommended to visualize hyphae. Despite the fact that microscopy can already provide important information (e.g., presence of septa, hyphal diameter, ramification pattern), a reliable differentiation between *Aspergillus* spp. and other filamentous fungi is impossible; in individual situations, although not standardized, and validated, immunohistochemical examination may be helpful (Hayden et al., 2003).

Although *Aspergillus* species are rarely recovered from the blood, all clinical samples from children who are at high risk for invasive fungal infections have to be cultured for fungi (Kontoyiannis et al., 2000). Blood samples and cerebrospinal fluid (CSF) should be processed immediately, and issue specimens have to be kept moist and must not be placed in fixatives. Importantly, negative culture results do not reliably exclude IA. If *Aspergillus* can be cultured, the isolate should be identified at the species level and antifungal susceptibility testing should be performed as this might impact therapy (Ruhnke et al., 2012).

## Fungal biomarkers

Due to the difficulties in isolating and culturing *Aspergillus*, there has been much effort to develop and evaluate various non-culture based assays. These assays include the serological detection of fungal antigens to support the diagnosis of IA in immunocompromised patients.

## Galactomannan

Galactomannan (GM) is a carbohydrate constituent of the cell-wall of *Aspergillus* spp. and is released by the fungus during cell growth. An FDA-approved enzyme immunoassay which employs the ß-1–5 galactofuranosyl specific EB-A2 rat monoclonal antibody has been evaluated in the clinical setting to detect the molecule (Platelia™ Aspergillus Enzyme Immunoassay, Bio-Rad). A number of studies evaluated the usefulness of GM as screening test in asymptomatic neutropenic adults (Maertens et al., 1999, 2001, 2004, 2005; Sulahian et al., 2001). Specificity and sensitivity of a positive test result were between 90 and 100% and 80 and 100%, respectively, and the negative predictive value (NPV) was greater than 90%. More importantly, positive test results were seen before patients developed clinical symptoms of IA or before abnormalities could be detected in a chest CT scan (Hayden et al., 2003). The EORTC/MSG consensus group included positivity of GM assessed in serum, BAL fluid and CSF in their revised definitions of IFD (De Pauw et al., 2008). In serum, a cut-off of 0.5 is recommended by the manufacturer, whereas a cut-off of ≥1.0 might be more appropriate for BAL fluid (Maschmeyer et al., 2015). In the adult setting, a number of guidelines recommend the use of GM for the diagnosis and management of IA (Maertens et al., 2010; Ruhnke et al., 2012; Maschmeyer et al., 2015).

A recent systematic review has scrutinized the available data of serum GM in pediatric cancer patients and children undergoing HSCT (Lehrnbecher et al., 2016). Eligible for the analysis were studies which included only patients up to 25 years of age, included at least 50% of patients suffering from cancer or undergoing HSCT, used EORTC/MSG criteria to define IA, and included patients without IA as a control group. The authors analyzed the test characteristics of 10 studies in which GM was used for screening and of eight studies which evaluated GM as a diagnostic tool, e.g., in patients with prolonged febrile neutropenia or in patients with pulmonary signs and symptoms. In the screening setting, a total of pediatric 688 patients were included, with the number of patients ranging between 17 and 198 per study; and in the diagnostic setting, 733 pediatric patients were included with a range of 38–145 patients per study. The prevalence of proven and probable IA ranged from 0.5 to 30.4 in the screening setting and from 0.0 to 30.8 in the diagnostic setting, respectively. Specificities and sensitivities ranged from 50 to 100% and from 0 to 100% in the screening setting and from 35 to 100% and from 14% to 100% in the diagnostic setting, respectively. Pooled specificity and sensitivity in the screening setting were 91% [95% confidence interval (CI), 86–94%] and 68% (95% CI, 51–81%), and 85% (95% CI, 51–97%), and 89% (95% CI, 79–95%) in the diagnostic setting, respectively. In adults, a meta-analysis has reported comparable data with a specificity of 81% (95% CI 72–90%) and a sensitivity of 82% (95% CI 73–90%) (Maertens et al., 2005). Positive predictive values (PPVs) in children were rather low and ranged between 0 and 100% in each setting, while the negative predictive values (NPVs) were considerably higher and ranging from 85 to 100% and from 70 to 100% in the screening and diagnostic setting, respectively. It is well known that there are a number of reasons for false-positive results, such as the concomitant use of some batches of beta-lactam antibiotics or the cross-reactivity of the assay with other fungi (e.g., *Penicillium* spp., *Fusarium* spp., *Histoplasma capsulatum, Cryptococcus neoformans*) (Viscoli et al., 2004). On the other hand, the use of mold-active prophylaxis may result in a high rate of false-negative results (Lass-Florl, 2017), although this could not be proven in the meta-analysis of pediatric studies as the antifungal prophylaxis was not standardized in most of the included studies. It is important to note that predictive values were similar when pediatric patients with possible IFD were classified as not having IFD or when patients with possible IFD were excluded from analysis. Similarly, predictive values were comparable in both screening and diagnostic testing setting, and test characteristics did not systematically improve as disease prevalence increased. Based on the data of this meta-analysis, recent recommendations of an international expert panel recommended to consider not using GM testing in children with prolonged febrile neutropenia (weak recommendation, moderate quality of evidence), because (1) due to the poor PPV, the clinical consequence based on test results is often incorrect (e.g., to institute mold-active antifungals) and (2) despite the high NPV, a negative GM test does not rule out non-*Aspergillus* molds, which clearly limits the value of the assay. However, as the diagnosis of IA is extremely difficult and does not depend on a single parameter alone, and as the local epidemiology plays an important role, GM testing may have an impact in daily clinical decision making with consideration of its limitations.

It is important to mention that the design of the available studies is in part highly problematic, as many used GM as the reference parameter to define IA. Similarly, in studies which required two positive samples to define a positive test result of GM, it often remains unclear during which time period the two samples had to be drawn. Another problem causing heterogeneity of the results of different studies is the variable aggressiveness to use CT scans or the invasive diagnostic procedures to prove or to exclude IA, all of which may bias the results.

Limited pediatric data are available on the assessment of GM in BAL. One study retrospectively analyzed the GM results in BAL fluid, which was obtained in 85 children (59 among them were immunocompromised) (Desai et al., 2009). Proven IA was diagnosed in three patients, probable IA in 6, whereas 39 children had possible IA and 39 no evidence of fungal disease. Receiver-operating characteristics demonstrated that in immunocompromised children, a BAL GM cut-off value of 0.87 resulted in a sensitivity, specificity, PPV and NPV for probable/proven IA of 78, 100, 58, and 96%, respectively. Although similar results were reported in another retrospective analysis of 72 bronchoscopies performed in immunocompromised children, the study demonstrated that the result of the GM assay in BAL had only little impact on the clinical decision, as in a considerable number of patients antifungal agents were continued or even started despite a negative GM result (de Mol et al., 2013); however, the duration of antifungal therapy was not considered in this analysis. Although colonization might be a bias for positive results of GM in BAL fluid, the promising results in children are supported by adult data on the use of GM testing in BAL fluid in patients in whom pulmonary aspergillosis was suspected (Maertens et al., 2009).

To date, relatively little data are published on the utility of GM testing in the CSF in order to diagnose CNS aspergillosis. A 18-months-old boy was reported with pulmonary and cerebral aspergillosis, in whom GM in the CNS was elevated (Roilides et al., 2003). In addition, one small case series reported on five patients with probable CNS aspergillosis (Viscoli et al., 2002). In these patients, GM levels in the CNS were significantly higher than GM levels of 16 control patients, suggesting the potential value of GM in CSF. Similarly, the testing of GM in urine specimens allows an easy and non-invasive sample collection, which would be interesting in particular in neonates and young children. In this respect, a recent study reported on promising results of measuring the urine GM/creatinine ratio in 71 adult patients with underlying hematological malignancies, but these results have to be validated in a pediatric patient population (Reischies et al., 2016).

Finally, there is growing interest in using serum GM as a surrogate marker for the effectiveness of antifungal therapy (Nouér et al., 2011; Chai et al., 2012; Kovanda et al., 2017). For example, one clinical trial in 158 adult patients demonstrated that an increase of the GM index of more than 0.25 from baseline by day 7 was associated with a significant increase of death compared to patients in whom the GM index increased less than 0.25 (Kovanda et al., 2017). Although not shown in the pediatric population, GM could individualize antifungal therapy as increases by day 7 could trigger treatment changes.

## 1-3-β-D-glucan

As compared to GM, the use of 1-3-ß-D-glucan (BG) as a biomarker allows the detection of a wider array of pathogenic fungi, including *Aspergillus* spp, *Candida* spp, *Fusarium* spp, *Trichosporon*, or *Pneumocystis jirovecii* since BG is present in the cell wall of many fungi. However, BG remains low or even negative in patients with cryptococcosis or mucormycosis. Positive values of BG can also be assessed during various bacterial infections, for example in infections due to *Streptococcus pneumoniae* or *Pseudomonas aeruginosa*, and additionally, BG may be detected in healthy individuals (Oz and Kiraz, 2011). Similar to GM, the EORTC/MSG consensus group included BG as mycological criterion in the revised definitions of IFD (De Pauw et al., 2008). A meta-analysis in adult patients demonstrated that different BG test assays have a similar performance [e.g., Fungitell™ (Associates of Cap Cod, Inc. Falmouth, MA), Fungitec-G (Seikagaku)], and that BG might be a useful tool to exclude IFD in the clinical setting, as for two consecutive tests, sensitivity and specificity were 50 and 99%, respectively, and estimated PPV and NPV for an IFD prevalence of 10% were 84% and 95%, respectively (Lamoth et al., 2012). However, it has to be noted that the assay has major limitations due to multiple factors which may cause false-positivity. For example, in addition to contamination, bacteremia, severe mucositis, the infusion of platelets with leukocyte-removing filters, the administration of albumin and immunoglobulins, or the administration of antibiotics such as amoxicillin-clavulanate or piperacillin-tazobactam have all been described as potential cause for false-positive results (Koltze et al., 2015). In addition, positive BG levels have to be interpreted with caution in patients who have had prior transient candidemia or have mucosal colonization with *Candida* species (Naselli et al., 2015).

There are limited data on the use of BG in children. It has been shown that mean values of BG are higher in normal healthy children than in healthy adults (Smith et al., 2007). It was suggested that the optimal cut-off level to identify neonates with invasive candidiasis was 125 pg/ml as compared to the 80 pg/ml recommended for adults, which resulted in a sensitivity and specificity of 84 and 75%, respectively (Goudjil et al., 2013). A total of three studies evaluated BG in children receiving therapy for a malignancy or undergoing HSCT, among them 38 patients with proven/probable IFD (Zhao et al., 2009; Badiee et al., 2012; Koltze et al., 2015). Overall, wide ranges were found for specificity (29–82%), sensitivity (50–83%), PPV (17–49%), and NPV (84–96%), respectively. Due to the limited pediatric data and the poor performance of the test in this patient population, BG testing is currently not recommended in the pediatric population as a routine diagnostic test, although it may be helpful under certain circumstances under awareness of its limitations (Groll et al., 2014; Lehrnbecher et al., 2017b).

## Molecular diagnostic tools

Polymerase chain reaction (PCR) has become a promising diagnostic tool for the early detection of IA. However, the method is not included to date as mycological criterion in the current EORTC/MSG definitions for IFD. This is due to a number of methodological issues, such as the optimal clinical sampling (e.g., whole blood, serum, plasma), the best DNA extraction method, and the optimal primer design (De Pauw et al., 2008; White et al., 2010, 2015b). In addition, multiple PCR approaches have been described, such as the use of nested PCR which detects genus-specific genomic sequences or single-copy genes, the use of “panfungal” PCR which detects multiple-copy genes identifiable in many fungal species and is followed by hybridization with species-specific probes, and real-time PCR technologies such as LightCycler™ or TaqMan™ which even may allow the quantification of fungal burden.

A total of 11 studies of PCR using blood samples of pediatric cancer patients were analyzed in a recent systematic review (Lehrnbecher et al., 2016). Three of the studies enrolling a total of pediatric 147 patients were performed in a screening setting, whereas 8 studies enrolling a total of pediatric 539 patients evaluated PCR samples in children with high suspicion of IA, such as children with prolonged febrile neutropenia or children with lung infiltrates. In the screening setting, specificity, sensitivity, PPV, and NPV ranged from 43 to 85%, 11 to 80%, 22 to 50% and 60 to 96%, respectively. In the diagnostic setting, specificity, sensitivity, PPV and NPV ranged from 36 to 83%, 0 to 100%, 0 to 71% and 88 to 100%, respectively. Due to these results, a recent international expert group gave a strong recommendation with a moderate quality of evidence for not using fungal PCR testing in blood (Lehrnbecher et al., 2017b). Whether the combined use of GM and PCR improves the test results as it was shown in a study in adult high-risk hematological patients needs to be evaluated in the pediatric population (Morrissey et al., 2013).

As compared to blood, the performance of PCR in BAL samples seems to be superior. For example, it has been demonstrated in 226 adult patients that BAL PCR testing had a sensitivity of 69% and a specificity of 87% in patients who did not receive antifungals prior to BAL (Reinwald et al., 2012). Unfortunately, the performance of the test significantly decreased in patients who had received at least two antifungals, which may be the situation in a considerable proportion of pediatric patients prior to receiving a BAL.

It is important to mention that PCR evaluation of biopsy samples may have an important impact in the correct diagnosis and in the treatment of pulmonary infections with a fungal pathogen, as it was demonstrated in adult patients (Lass-Flörl et al., 2007). In addition, PCR based methods have also been investigated and are of major usefulness for the identification of mutations conferring resistance to specific antifungals (e.g., azole resistance in *A. fumigatus*) (White et al., 2015a).

## Imaging studies

As autopsy data demonstrate that the lung is affected in almost 90% of the patients with IA (Groll et al., 1996; Lehrnbecher et al., 2010), imaging of the lung is an important tool in the early diagnosis of the infection. In adults, chest computerized tomography (CT) scans reveal pneumonia due to a fungal pathogen earlier as conventional chest radiographs (Heussel et al., 1999), which is important in order to institute early antifungal therapy which impacts upon outcome. In adults, CT findings such as the “halo sign,” the “air-crescent sign”, or “cavitation” have been included in the current EORTC/MSG definitions of IFD (Caillot et al., 1997; De Pauw et al., 2008). Nevertheless, these findings are not specific for pulmonary aspergillosis and may also be seen in other pulmonary infections or due to the progression and relapse of the underlying malignancy. In addition, the appearance of these signs depends on the time of imaging (Caillot et al., 1997).

Data on CT findings in children with IA are conflicting. One retrospective study in 139 children with IA demonstrated that the halo sign was seen in only 6.4% of the patients, the air-crescent sign in 1.6% of children and a cavitation in 14.4% of patients, respectively (Burgos et al., 2008). In contrast, nodules were found in 21% of the pediatric patients, corroborating the results of another report (Taccone et al., 1993). In contrast, a more recent pediatric study found that the halo sign in the chest CT was significantly associated with pulmonary IA (78.4 vs. 40.7%, *P* < 0.001) (Han et al., 2015). It is unclear whether the different results among the studies simply reflect different time points of CT imaging or are due to other, yet unknown reasons. Nevertheless, the appearance of any new infiltrate in the chest CT in children with prolonged febrile neutropenia not responding to broad-spectrum antibiotics should be considered as possible pulmonary fungal disease and trigger diagnostic procedures. Notably, in adults, high resolution CT angiography may improve radiological diagnosis by detecting vessel occlusions caused by invasive aspergillosis, but to date, no data are available in the pediatric population (Stanzani et al., 2012, 2015).

Regarding the radiologic evaluation of other sites in children with prolonged febrile neutropenia and a high suspicion of IFD, a recent pediatric guideline included a weak recommendation for routine abdominal imaging, as in four studies, imaging findings in a significant proportion of patients without localizing signs or symptoms were consistent with IFD (Bartley et al., 1982; Archibald et al., 2001; Ahmad Sarji et al., 2006; Cohn et al., 2016; Lehrnbecher et al., 2017b). In contrast, the guideline panel gave a weak recommendation against routine sinus imaging in persistently febrile neutropenic patients in the absence of localizing signs or symptoms, which is based on the observation that sinus imaging is frequently abnormal in these patients and that the findings do not distinguish between children with and without invasive fungal infection of the sinus (Kavanagh et al., 1991; Archibald et al., 2001; Park et al., 2005; Ahmad Sarji et al., 2006; Cohn et al., 2016; Lehrnbecher et al., 2017b).

Whereas the lung is the primary portal of entry of *Aspergillus*, an autopsy study demonstrated that the infection is confined to the lung in only 41%, but disseminated in 53% of the patients, and that the CNS is one of the most common sites of disseminated involvement (Groll et al., 1996). The clinical presentation of cerebral aspergillosis, which is associated with an extremely high mortality, depends on the site and extent of involvement and can be classified in a number of defined syndromes, such as fungal abscesses, granulomas, or cavitated lesions (Armenian et al., 2009; Castagnola et al., 2010). For these patients, magnetic resonance imaging (MRI) of the CNS is the most important diagnostic tool in order to identify and monitor cerebral fungal infections. There is an ongoing debate whether MRI of the CNS should routinely be performed in asymptomatic patients with pulmonary aspergillosis.

## Invasive diagnostic procedures

Invasive diagnostic procedures such as bronchoscopy combined with BAL or lung biopsy should be considered in the work-up of children with pulmonary infiltrates. Whereas, the isolation of *Aspergillus* spp. from the sputum of neutropenic patients with clinical symptoms consistent with IFD is only an indicator of fungal pneumonia, the histopathological identification and/or the culture of *Aspergillus* spp. from lung tissue remains the diagnostic “gold standard” for proof of pulmonary aspergillosis (Ruhnke et al., 2012).

A recent meta-analysis on the diagnostic yield and the complication rate of BAL and lung biopsies performed in both pediatric and adult patients with cancer or HSCT recipients included 72 studies on BAL and 31 studies on lung biopsies (Chellapandian et al., 2015). When comparing the two diagnostic tools, the proportion of procedures leading to any diagnosis including non-infectious diagnoses was similar. However, BAL led significantly more often to an infectious diagnosis than did lung biopsy (49 vs. 34%; *P* < 0.001). A significant difference was seen for the identification of a bacterial infection, but not for the detection of viral and fungal infection, respectively. In contrast, lung biopsies resulted significantly more often in a non-infectious diagnosis than BAL (43 vs. 7%; *P* < 0.001), but, at the same time, was associated with a significantly higher complication rate (15 vs. 8%; *P* = 0.006). Whereas the diagnostic yield of BAL and lung biopsy seems to be similar between the pediatric and adult population, children had a higher complication rate during biopsy procedures than adults (*P* = 0.003). Studies which included the assessment of GM in BAL reported on a significantly higher detection rate of proven/probable IA than studies in which GM was not used (31%; 95% CI, 23–43% vs. 18%; 95% CI, 15–22%; *P* = 0.005), whereas the impact of PCR as a diagnostic tool in BAL samples is less clear. Notably, as mentioned above, the diagnostic performance of the PCR in BAL samples decreased in adult patients who had received at least two antifungal agents (Reinwald et al., 2012). In contrast, the diagnostic yield of invasive diagnostic procedures increased when different diagnostic tools (e.g., histopathology, antigen- and molecular testing) were combined (Lass-Flörl et al., 2007). On the other hand, according to a prospective study in 55 adult patients, a positive mycologic result in respiratory samples depended on the underlying malignancy and the leukocyte count, with significantly higher yields in non-acute leukemia and in patients with leukocyte counts of more than 100 per μl (Bergeron et al., 2012). A meta-analysis showed that results of lung biopsies led significantly more often to a change in the clinical management (48% vs. 31%; *P* = 0.002), but it remains unclear whether this was associated with an improved overall outcome in this highly selected patient population.

## Summary and future perspectives

The early and reliable diagnosis of IA is difficult in immunocompromised patients, in particular in the pediatric population. In high risk patients with prolonged febrile neutropenia or signs and symptoms for IA, available diagnostic tools include imaging procedures and non-culture based techniques such as antigen-based and molecular biomarkers. The performance and clinical utility of these tests may be different between children and adults, and they may also be different for invasive diagnostic procedures such as BAL or biopsy. However, it is important to note that considerably less data are available for children as compared to adults. Although the combination of diagnostic methods seems to improve sensitivity and specificity, new diagnostic tools are needed that will improve the early and reliable diagnosis of IA. In this respect, the lateral-flow device assay which is based on the JF5 antibody and detects an extracellular glycoprotein antigen secreted during active growth of *Aspergillus* spp, is a promising platform that is to be tested in the pediatric population, as it may allow for a rapid and simple bed-side testing of IA (Heldt and Hoenigl, 2017; Hoenigl et al., 2018). In addition, specific host response biomarkers may be an interesting approach to diagnose IA, as it has been demonstrated for the monitoring of *Aspergillus*-specific CD4^+^ T-cells (Bacher et al., 2015). Another interesting approach is the use of *A. fumigatus-*specific antibodies labeled with a radionuclide, which can be visualized by Positron Emission Tomography (PET)/MRI. Although not tested yet in humans, this approach could clearly distinguish invasive pulmonary aspergillosis from bacterial lung infection and other non-specific lung inflammation in the mouse model (Rolle et al., 2016). However, in order to perform meaningful studies to evaluate these diagnostic tools in the pediatric population, a comprehensive and necessarily international effort is necessary.

## Author contributions

TL, AH, AG, and KB designed the manuscript, wrote parts of the manuscript, reviewed, and approved the last version of the manuscript.

### Conflict of interest statement

The authors declare that the research was conducted in the absence of any commercial or financial relationships that could be construed as a potential conflict of interest. The reviewer VS and handling Editor declared their shared affiliation.
